# Effects of Part- and Whole-Object Primes on Early MEG Responses to Mooney Faces and Houses

**DOI:** 10.3389/fpsyg.2016.00147

**Published:** 2016-02-16

**Authors:** Mara Steinberg Lowe, Gwyneth A. Lewis, David Poeppel

**Affiliations:** ^1^Department of Communicative Sciences and Disorders, New York UniversityNew York, NY, USA; ^2^Department of Psychology, New York UniversityNew York, NY, USA; ^3^Department of Neuroscience, Max-Planck-Institut für empirische ÄsthetikFrankfurt, Germany

**Keywords:** MEG, mooney, face recognition, object recognition, M100, M170

## Abstract

Results from neurophysiological experiments suggest that face recognition engages a sensitive mechanism that is reflected in increased amplitude and decreased latency of the MEG M170 response compared to non-face visual targets. Furthermore, whereas recognition of objects (e.g., houses) has been argued to be based on individual features (e.g., door, window), face recognition may depend more on holistic information. Here we analyzed priming effects of component and holistic primes on 20 participants' early MEG responses to two-tone (Mooney) images to determine whether face recognition in this context engages “featural” or “configural” processing. Although visually underspecified, the Mooney images in this study elicited M170 responses that replicate the typical face vs. house effect. However, we found a distinction between holistic vs. component primes that modulated this effect dependent upon compatibility (match) between the prime and target. The facilitatory effect of holistic faces and houses for Mooney faces and houses, respectively, suggests that both Mooney face and house recognition—both low spatial frequency stimuli—are based on holistic information.

## Introduction

Stimulus selectivity is a major focus in studies of face and object perception. While object recognition has been argued to be based on featural representations (e.g., doors and windows represent houses; cf. Tanaka and Farah, 1993), face recognition may involve holistic/configural information (Maurer et al., 2002). “Featural” refers to individual components (e.g., nose size), while “configural” refers to relations between components. Maurer et al. (2002) distinguish configural processes as *first-order relational—*(structure/arrangement of features, e.g., eyes above mouth), *holistic*—(merger of features, e.g., a gestalt), and *second-order relational*—(spacing between features). Natural face recognition may involve all three stages whereas manipulated images of faces may recruit theses putative operations in more limited ways. Schematic faces (e.g., smiley faces) lacking meaningful physiognomic information should not engage second-order processing (Sagiv and Bentin, 2001) whereas two-tone Mooney faces lacking first-order features that are individually recognizable must be recognized holistically (Latinus and Taylor, 2005).

Much of the research on face perception has focused on the kinds of information people extract when viewing an upright face. While it has been suggested that face perception utilizes both holistic and featural information (e.g., Maurer et al., 2002), it is generally thought that holistic information plays a greater role (e.g., Tanaka and Farah, 1993; Farah, 1996; Rossion et al., 1999). The “holistic-view” has been supported by the finding of certain phenomenon only observed for faces. The face inversion effect is the robust finding of severely hindered recognition of upside-down faces relative to upright faces, which is not observed for other objects (e.g., Maurer et al., 2002). This suggests that upright faces recruit a more efficient process that extracts information as a combined entity, whereas inverted faces (and non-faces) involve slower “piecemeal” analyses of individual features.

Whether such phenomena support face recognition as primarily holistic is challenged by results from numerous vision research studies (e.g., Sekuler et al., 2004; Konar et al., 2010; Gold et al., 2012). In one of these studies, participants viewed individual faces embedded in noise, and then selected a matching face from two noise-free faces of varying contrast (Sekuler et al., 2004). They employed a response classification technique to identify influential regions of the images on discrimination, and also quantified the amount and efficiency of the information used in discrimination. The major finding was that although the same amount of information from the same localized regions were used, the level of efficiency was much higher for upright than inverted faces. This finding suggests that the face-inversion effect, commonly taken as evidence for holistic face perception, can instead be explained in terms of quantitative shifts, where the utility of upright information is simply more efficient than inverted information (Sekuler et al., 2004). Further support for quantitative accounts of the face-inversion effect comes from Gold et al. (2012). They compared recognition performance for whole faces with that predicted by an optimal Bayesian integrator (based on recognition performance for isolated components). Simply put, Gold et al. found that performance for both upright and inverted faces was the same as (or even worse than) the summed performance of the parts. Furthermore, they found that lower integration performance for inverted than upright faces, which further suggests that the face-inversion effect reflects quantitative rather than qualitative differences.

Another robust effect often considered to support the holistic-view is the composite face effect (CFE), first introduced by Young et al. (1987). In a CFE task, participants view two combined halves of different faces, ignore one of the halves, and make judgments about the other half. The halves are aligned in one condition, and misaligned in another. The CFE is calculated by subtracting the performance for the misaligned halves from performance for the aligned halves. A robust finding is that performance is worse in the aligned condition (Young et al., 1987; Hole, 1994). Presumably, aligned halves appearing to be a single object were processed holistically, leading to interference from the irrelevant half. The CFE for inverted faces is relatively weaker, presumably because they are analyzed on the basis of individual features (Young et al., 1987; Hole, 1994). Using CFE as an index of holistic processing, Konar et al. (2010) hypothesized that if holistic processing is important for face perception, then individual differences in face identification accuracy should correlate with individual differences in CFE magnitude. They found that although CFE varied considerably across individuals, differences in magnitude were not correlated with individual differences in accuracy. Later findings from Richler et al. (2011a,b) suggest that the presence or absence of holistic processing effects depends on the choice of holistic-processing measure. Using a revised version of of Konar et al.'s (2010) composite face task that accounted for possible response bias, Richler et al. found evidence of holistic processing not just for upright faces (Richler et al., 2011a) but also inverted faces (Richler et al., 2011b).

A number of neurophysiology experiments have addressed the nature of face perception. Results from cognitive neuroscience, particularly EEG and MEG studies, provide evidence for face-selective mechanisms, reflected in occipitotemporal response patterns. Strongly implicated is the M170 (or its EEG counterpart, N170). Faces elicit greater amplitude than non-faces when presented as photographs (Halgren et al., 2000), line drawings (Liu et al., 2000), or Mooneys (Latinus and Taylor, 2005). Moreover, natural-face inversion elicits delayed, enhanced N170 amplitude (Sagiv and Bentin, 2001), which is also observed for Mooney faces, but only after initial training (Latinus and Taylor, 2005).

Another class of studies investigates face and object recognition with adaptation paradigms and associated repetition effects, i.e., repeated stimulus presentation to identify neural substrates supporting, for example, face processing. In this context, response invariance is inferred when adaptation to one stimulus is the same as for two stimuli differing in one dimension (e.g., orientation). Harris and Nakayama (2007) examined M170 adaptation to natural faces as a function of non-face vs. face attenuators of different forms, i.e., as photos, line-drawings, and Mooneys. Stronger adaptation from face-attenuators of any type suggested that low-level visual features do not explain M170 face-selectivity. Subsequently, Harris and Nakayama (2008) examined configural vs. featural influences on M170 face adaptation, finding stronger face adaptation when attenuators were upright or inverted faces, face components (e.g., nose), or non-face configurations of face-parts; notably, weaker adaptation attenuators were face-configurations comprised of non-face parts, suggesting that configuration does not explain M170 face-selectivity.

The M100 response (or its EEG counterpart, P1) has been shown to discriminate faces from non-faces, but may be more sensitive to sensory properties of stimuli than “faceness” or subtle changes between components (Halgren et al., 2000), suggesting a more preliminary role in face recognition. Additionally, Liu et al.'s (2002) study of superordinate- and subordinate-levels of face categorization found that while M170 indicated involvement in both face detection and identification (i.e., discriminating one person's face from another), the M100 seemed limited to face detection. Previous work presents conflicting evidence regarding face-inversion effects at P1. Rossion et al. (1999) failed to find face-inversion effects on P1, presumably because inversion does not disrupt low-level visual features at a relevant scale. Other work, however, reports typical P1 inversion effects elicited by non-degraded faces (N170 inversion effects were elicited by both non-degraded and degraded faces; Latinus and Taylor, 2005). Still more recent work found earlier P1 for faces, as well as enhanced and delayed P1 for inverted than for upright faces (Itier and Taylor, 2004). Note that all of these studies employed natural face stimuli. To our knowledge, it has yet to be reported that Mooney faces (even after training) induce changes in P1/M100 amplitude (George et al., 2005; Latinus and Taylor, 2005, 2006), suggesting it is not sensitive to holistic information of Mooneys.

Priming effects on the N170 during recognition of familiar but not unfamiliar Mooney faces are consistent with the view that top-down knowledge penetrates early perceptual stages (Jemel et al., 2003, 2005). Mooney faces, which are ambiguous and difficult to recognize, may lead to greater reliance on top-down knowledge. Pre-activated structural representations (component vs. holistic) may influence ambiguous image recognition, depending on whether the relevant processes operate on holistic or component properties. An ERP priming experiment by Jemel et al. (2005) examined N170 responses to Mooney images of famous and non-famous people in three priming conditions: (1) Same-person prime: photo or name of the same person, (2) Unrelated prime: photo or name of a different person, (3) Neutral: white oval or string of X's. Jemel et al. found enhanced amplitude for famous (but not non-famous) faces when preceded by the same face or a corresponding name. The finding of priming both within-domain (same photo) and cross-domain (corresponding name) together with the lack of repetition effects on the N170 for non-famous people, suggest that the N170 reflects top-down processing (see also Jemel et al., 2003) rather than just bottom-up visual input.

We hypothesized that if faces are recognized holistically, pre-activated holistic structures should facilitate recognition, as reflected, for example, by decreased M170 latency or amplitude. We thus conducted a priming experiment in the context of MEG recording to examine effects of configural/holistic and featural/component primes on occipitotemporal responses to Mooney targets. Holistic primes were individual faces and whole houses, while component primes were isolated face-parts (e.g., nose) and house-parts (e.g., door). Greatest facilitation derived from whole-face primes, as reflected in decreases in M170 amplitude and latency, would indicate holistic processing. Greatest facilitation from face-part primes would indicate featural processing. As for houses, if components ultimately represent non-faces/objects, part-house primes should maximally prime recognition of Mooney houses.

## Materials and methods

### Participants

Twenty-five adults (15 females, mean age 29 years), with normal or corrected-to-normal vision, from the New York University community participated after providing written informed consent in accordance with the University's Committee on Activities Involving Human Subjects. Five participants were excluded due to excessive artifacts, equipment failure, or missing data. Analyses thus included data from 20 participants.

### Stimuli

#### Primes

**Figure 1:**
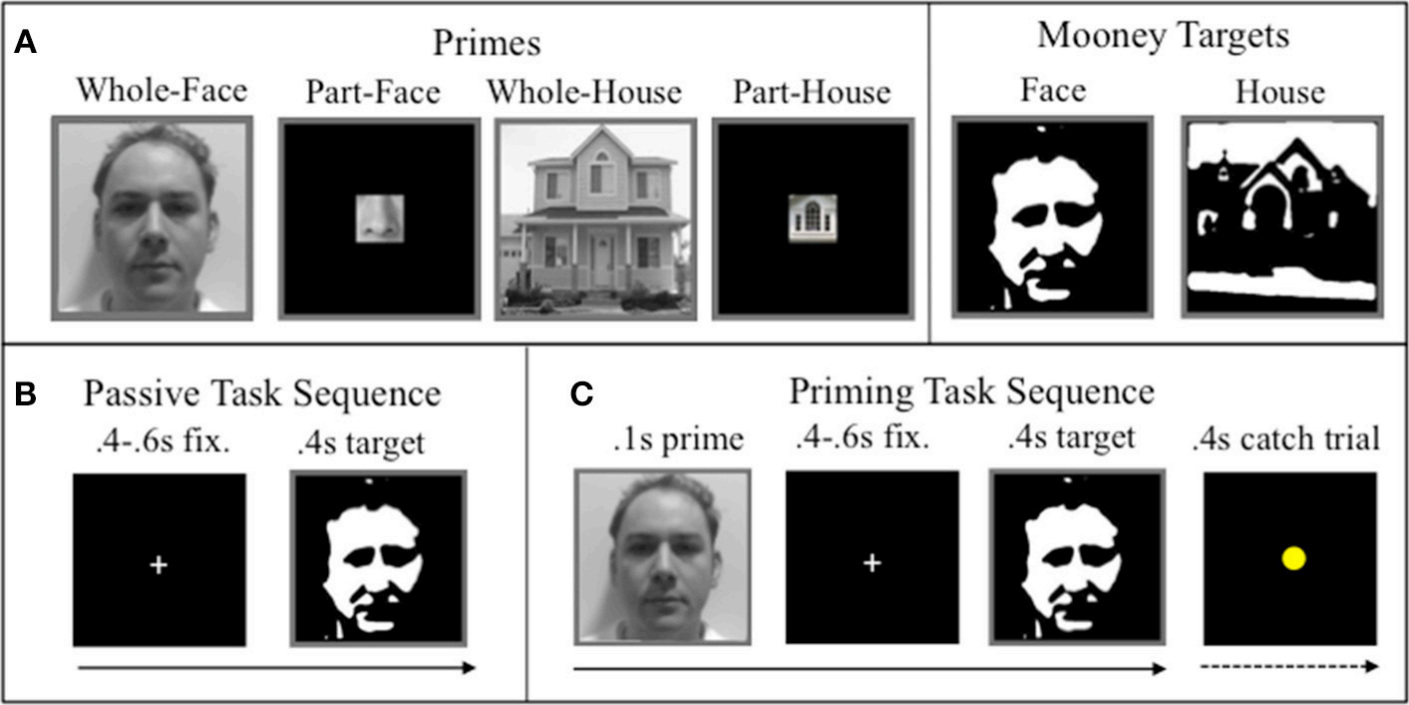
**Examples of (A) stimuli, (B) passive-task trial-sequence, (C) priming-task trial sequence**.

Four kinds of primes (40 each) were created from gray-scale unfamiliar images obtained from Internet searches (e.g., Google Images): whole-face, part-face, whole-house, and part-house (Figure 1A). Face images showed frontal views of adult males of various ethnicities, of neutral or pleasant expression, and without facial hair, glasses, hats, or accessories. The house images showed unobstructed frontal views of traditional houses from a variety of architectural styles (e.g., colonial, ranch). House and face images were isolated from their background and cropped to create the four different kinds of primes. Whole-face primes were isolated from their backgrounds and only included faces from the neck up, while part-face primes showed isolated facial features (nose, mouth, ear, or eyes). Whole-house primes were also isolated from their background to showed buildings in their entirety while excluding most of the surrounding scenery. Part-house primes were cropped to isolate individual building features (window, door, chimney, or roof). Whole primes were resized to 300 × 300 pixels (72 dpi), while part primes were resized to 75 × 75 pixels and then centrally enclosed in a black square (300 × 300 pixels). The resized gray-scale images were all enclosed in a gray border to frame the visual space. All stimuli were presented on a gray background.

#### Mooney targets

A separate set of 40 houses and 40 faces were similarly obtained and subsequently transformed into Mooney images with MATLAB functions (MathWords, Inc., Natick, MA, USA). First, “fspecial” (hsize = 30, sigma = 25) created a two-dimensional Gaussian filter. Next, “imfilter” convolved the filter with each image. Last, “imbw” converted the images to black-and-white Mooneys. All primes and targets were resized to 300 × 300 pixels (72 dpi) and framed by the same gray border as the primes (Figure 1A). A separate set of Mooney images (from new images of faces and houses) were also generated for use in a separate task.

### Experimental procedure

Each participant's head-shape, fiducials, and head-position indicator coil locations were digitized prior to the experiment. Participants lay supine on a scanner bed in a sound-attenuated, magnetically-shielded room. Participants passively listened to tones (1000 Hz, 70 dB SPL, *n* = 100) with closed eyes in an auditory localizer control task.

Stimuli were presented with MATLAB Psychtoolbox in pseudorandomized order. Images were presented on a gray screen 44 cm above the face. Larger stimuli, including the whole primes and Mooney targets, subtended ~9.1° while part primes subtended ~2.3°. Participants performed a passive viewing task of Mooney faces and houses to obtain baseline neural responses. Trials in the passive viewing task commenced with a 500–900 ms fixation point (+), followed by a 400 ms target (i.e., Mooney face or Mooney house). Participants were instructed to passively but attentively view the images. Participants viewed a total of 320 Mooney images (80 faces and 80 houses, each presented twice) over the course of two blocks. This part of the experiment lasted approximately 6 min. Figure 1B shows the trial sequence of the passive task.

In the priming task, neural responses to target Mooney images were assessed as a function of different primes (wholeness vs. partness, same vs. different category). Figure 1C shows the trial sequence. To ensure visual attention, participants were instructed to press a button when a yellow dot appeared (15% of the trials). Trials in the priming task consisted of the following sequential events: a 100 ms prime, a 400–500 ms fixation point, a 400 ms target Mooney stimulus, and finally, a 600 ms fixation point that signaled the end of the trial. There were a total of 160 unique primes (40 each of the 4 prime types) and 160 unique Mooney targets (80 houses, 80 faces). Primes were each paired with two Mooney faces and two Mooney houses. Likewise, each target was paired with two of each prime types. Primes were therefore repeated four times and targets were repeated eight times over the course of four blocks (with order shifted so that the same prime did not appear in two sequential blocks). The task consisted of 640 total trials and lasted ~20 min.

### MEG recording

A 160 axial gradiometer (157 data channels, 3 reference channels) whole-head system (Kanazawa Institute of Technology, Kanazawa, Japan) continuously acquired MEG data (500 Hz sampling rate, 60 Hz band-reject filter, DC recording). Noise-reduction used data from three reference sensors by way of the Continuously Adjusted Least-Squares Method (CALM; Adachi et al., 2001). Trials were epoched into 500 ms events (100 ms pre-stimulus onset), averaged, baseline corrected using the 100 ms pre-stimulus interval, and low-pass filtered (30 Hz cutoff).

### Analysis

A template waveform of participants' grand-average priming data across all priming conditions and trials informed identification of time-windows^1^ around the M100 peak (*M* = 125 ms, *SD* = 15) and the M170 peak (*M* = 202 ms, *SD* = 22). The time windows were centered around the peaks and included approximately ±2 standard deviations from the mean (M100: 80–160 ms; M170: 160–240). Participants' amplitudes and latencies were calculated for the M100 and M170 peaks. Because the relative changes in amplitude and latency of each peak in relation to the prime were of interest in this study, a normalization procedure was applied to account for individual differences across participants. Normalization was based on the average of the values within a given response (i.e., M100 amplitude, M100 latency, M170 amplitude, or M170 latency). To do this, the average of each participant's peak amplitude or latency across all prime conditions per response was calculated and subtracted from each prime condition in that response.

Passive task data were analyzed in four separate paired *t*-tests; for latency and amplitude of each response. The normalized priming task data were analyzed in four separate linear regression models (*lm* function) in R (http://www.r-project.org/) for latency and amplitude of each response. Analyses were only on waveform peak responses to Mooney targets and not primes.

A multiple linear regression model was created to predict each response (e.g., M170 amplitude) based on the following independent variables: (1) Target: Mooney Face vs. Mooney House, (2) Prime Wholeness: whole-prime vs. part-prime (averaged across face and house primes), and (3) Prime/Target Match: face-prime → Mooney face; house-prime → Mooney house; face-prime → Mooney house; house-prime → Mooney face. In light of the design and hypotheses, the full model also included the following interactions: (1) Target × Prime Wholeness, (2) Target × Prime/Target Match, (3) Prime Wholeness × Prime/Target Match, and (4) Target × Prime Wholeness × Match.

Log-likelihood comparisons identified which interactions significantly contributed to the final models chosen for each dependent variables (Baayen, 2008; Baayen et al., 2008). *Post-hoc* pairwise comparisons were conducted using Least Squares Means (*lsmeans* function; Lenth, 2015) and Tukey's adjustment to interpret significant interactions. Full model outputs are provided in the Supplementary Materials (Table S1).

A bootstrap procedure was used to further assess significant differences between conditions across the participants (similar to methods used in Rousselet et al., 2008). First, the mean was calculated across participants for each condition independently (see Figure S6). Then mean differences between the contrasts of interest were calculated (e.g., Part Match vs. Whole Match). The functions *bootci* and *bootstrp* in MATLAB were used to compute a sample of 1000 bootstrapped means and to create a confidence interval (alpha = 0.05) around each mean difference. At each resample, the group mean was computed, resulting in a distribution of resampled means for computing the SEM (Figure S7) and confidence intervals (Figure S8).The difference between means was considered significant if the 95% confidence interval did not include zero.

## Results

**Figure 2:**
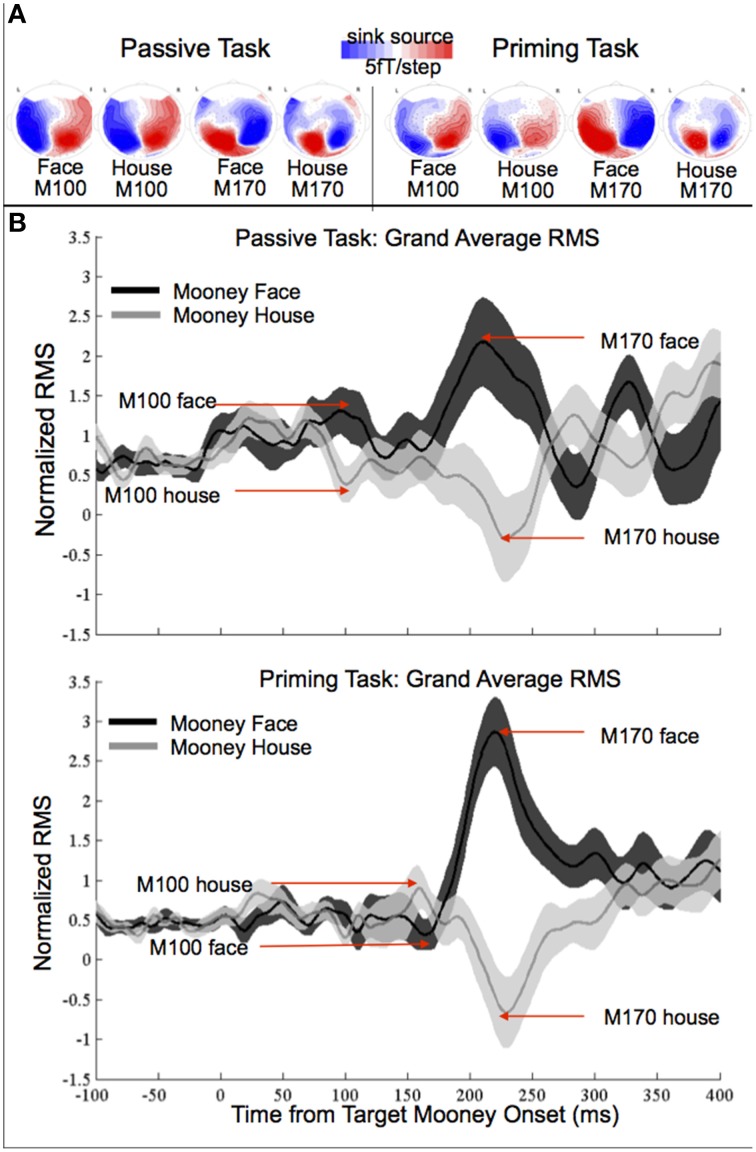
**(A)** Grand-average contour maps of neuromagnetic response distributions of M100 and M170 components in passive- (left) and priming-tasks (right). **(B)** Grand-average root-mean-squares (RMS) of normalized MEG activation (106 sensors) and standard error in passive and priming tasks.

Contour maps and the normalized root mean square (RMS) waveforms of the grand average M100 and M170 responses are shown in Figure 2 (non-normalized data are shown in Figure S1). We first present the results for the passive task, followed by the results for the priming tasks. The final regression models for the priming task included Target, Prime Wholeness, Prime/Target Match and any significant interactions (as described above). Interactions that did not significantly contribute to a given model were excluded and are therefore not described.

In the passive task, the amplitude was significantly greater for faces than houses in both the M100 [*t*_(17)_ = 2.28, *p* = 0.036, *d* = 0.38], and M170 responses [*t*_(17)_ = 2.77, *p* = 0.013, *d* = 0.64]. There was no significant difference in latency between houses and faces for either response (all *p*s > 0.05).

### M100 results

M100 latency was not significantly associated with Target, Prime Wholeness, Prime/Target Match or the two-way interactions (all *p*s > 0.05). Although the three-way interaction was significant by conventional criteria (*p* = 0.049), *post-hoc* tests revealed no significant differences between contrasts (all *p*s > 0.05).

**Figure 3:**
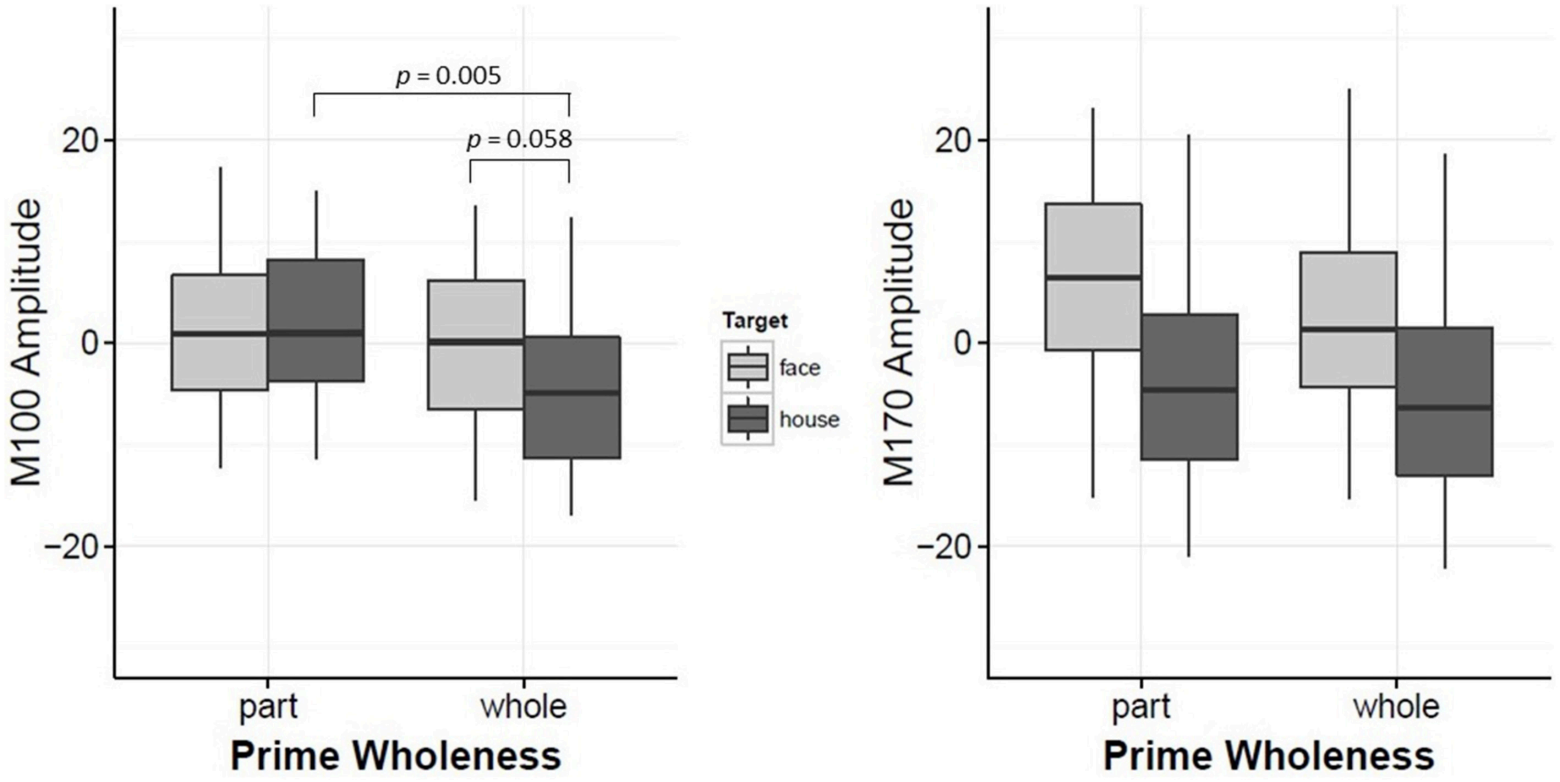
**Plots for M100 Amplitude and M170 Amplitude interaction between Prime-Wholeness and Target**. Horizontal lines show the median, box edges show the first and third quartiles. Significant results are marked in the figure.

**Figure 4:**
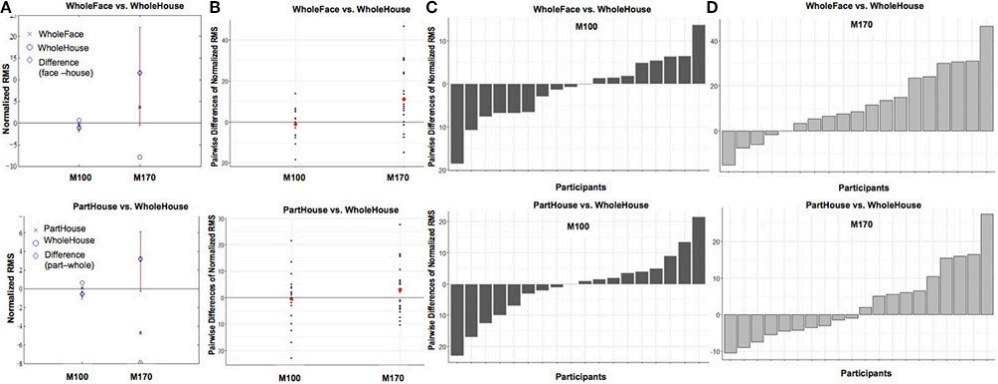
**Comparisons of target (face vs. house) when preceded by a whole prime (A) and of prime wholeness (part vs. whole) when followed by a Mooney House (B)**. For each condition comparison, Column **(A)** shows mean difference between the conditions, plotted with a diamond, and the confidence interval (percentile bootstrap, 1000 sample trials, *p* < 0.05) around the mean difference marked with a red line. The mean difference is significant when the confidence interval does not contain zero. Column **(B)** shows the scatterplot of the pairwise differences for each condition comparison. Each black dot represents an individual participant. The mean response difference is represented by a red dot. The pairwise difference for each participant in each condition comparison is shown for the M100 response in Column **(C)** and the M170 response in Column **(D)**.

Target led to significant changes in the M100 amplitude, such that the overall amplitude for Mooney faces was significantly greater than Mooney houses (β = −4.84, *t* = −2.54, *p* = 0.012, *d* = 0.40). However, this effect was modulated by Prime Wholeness (i.e., the interaction between Target and Prime Wholeness was significant, β = −5.62, *t* = 2.12, *p* = 0.036; Figure 3 and Figure S4). *Post-hoc* testing revealed that the M100 amplitude was greater in response to Mooney faces than Mooney houses only when the target was preceded by a *whole prime;* but this effect was not significant in the regression analysis (*p* = 0.058, *d* = 0.03). Using the bootstrap procedure, the confidence interval for M100 amplitude in this comparison did not contain zero and therefore the mean difference was considered significant (Figure 4A). The M100 amplitude did not differ in response to Mooney faces and Mooney houses when preceded by a *part prime* (*p* = 0.975; Figure 3). Additionally, the M100 amplitude for Mooney houses was significantly greater in part-prime trials than in whole-prime trials (e.g., part-face/part-house → Mooney house > whole-face/whole-house → Mooney house; *p* = 0.005, *d* = 0.29; Figure 3;). Fifteen out of 20 participants showed the effect in the group direction (Figure S2). This pairwise difference was also significant based on the bootstrap analysis (Figures 4B–D).

M100 amplitude was not significantly associated with Prime Wholeness, Prime/Target Match or the interaction between Prime Wholeness and Prime/Target Match (all *p*s > 0.05).

### M170 results

Target led to significant changes in M170 latency such that the response was later for Mooney faces (*M* = 206, *SD* = 59) than Mooney houses (*M* = 198, *SD* = 72; β = −6.92, *t* = −4.572, *p* < 0.001, *d* = 0.80).

Target was associated with a significantly stronger M170 amplitude for Mooney faces than Mooney houses (β = −9.44, *t* = −5.61, *p* < 0.001, *d* = 0.31). Prime/Target Match also led to significant changes in M170 amplitude (β = −8.44, *t* = −3.55, *p* < 0.001, *d* = 0.19). Mismatched prime-target trials (e.g., face prime → Mooney house) resulted in significantly stronger M170 amplitude relative to matched trials (e.g., face prime → Mooney face).

**Figure 5:**
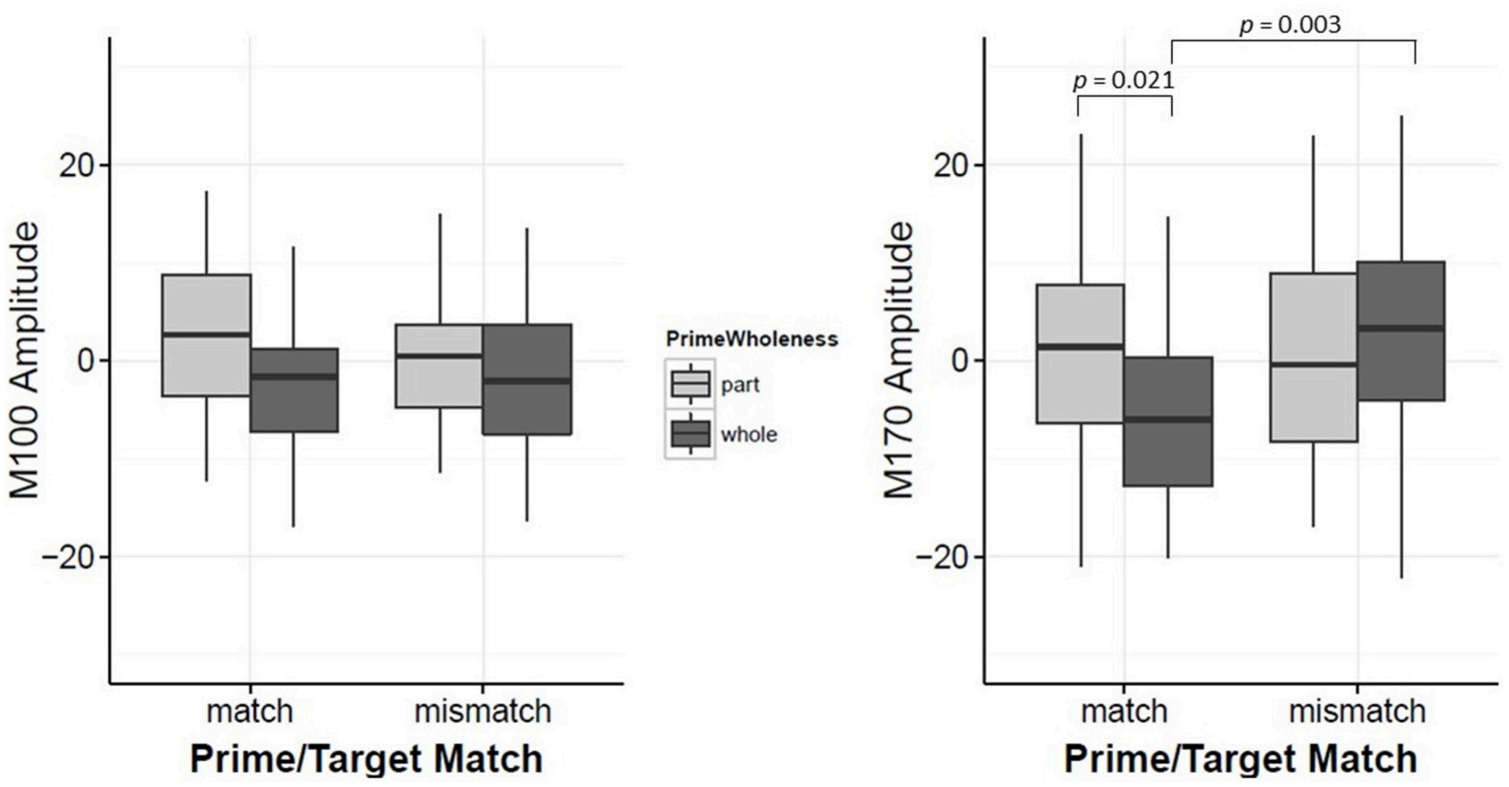
**Plots for M100 Amplitude and M170 Amplitude interaction between Prime-Wholeness and Prime-Target-Match**. Horizontal lines show the median, box edges show the first and third quartiles. Significant results are marked in the figure.

**Figure 6:**
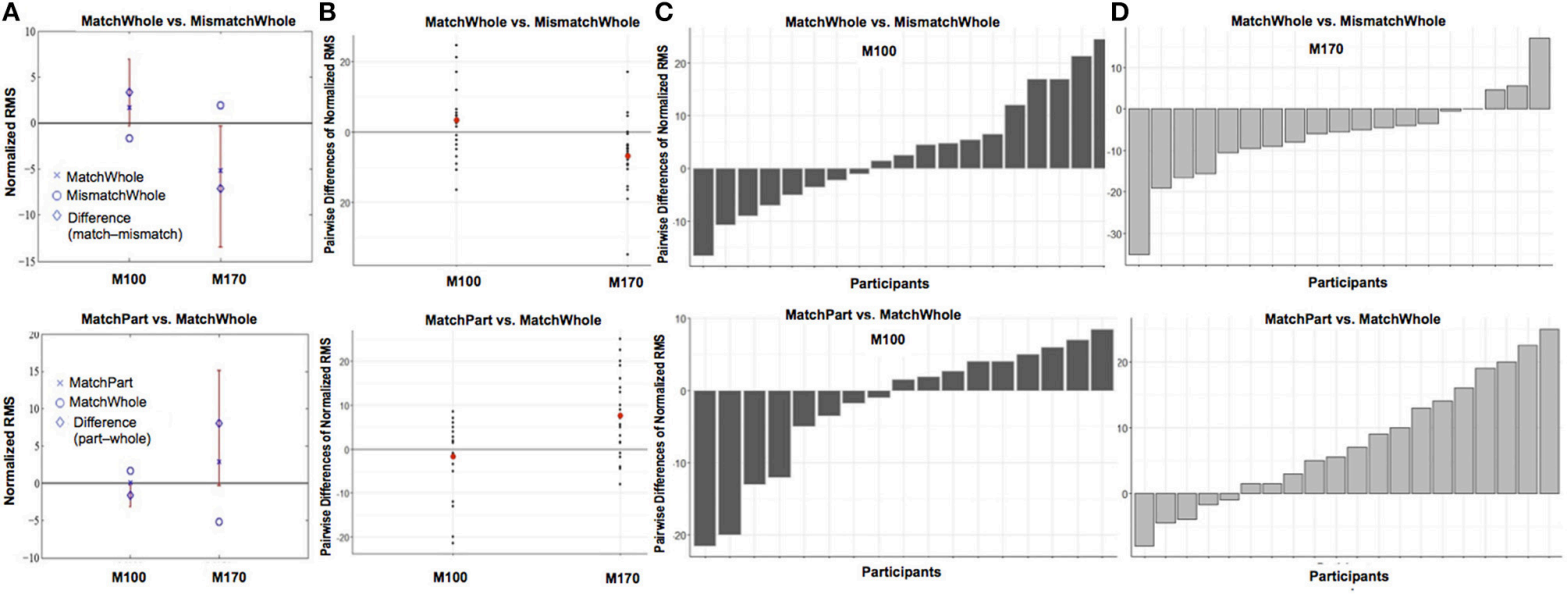
**Comparisons of prime wholeness (part vs. whole) when the prime and the target were matched (A) and of prime/target matched when the prime was whole (B)**. For each condition comparison, Column **(A)** shows mean difference between the conditions, plotted with a diamond, and the confidence interval (percentile bootstrap, 1000 sample trials, *p* < 0.05) around the mean difference marked with a red line. The mean difference is significant when the confidence interval does not contain zero. Column **(B)** shows the scatterplot of the pairwise differences for each condition comparison. Each black dot represents an individual participant. The mean response difference is represented by a red dot. The pairwise difference for each participant in each condition comparison is shown for the M100 response in Column **(C)** and the M170 response in Column **(D)**.

While Prime Wholeness alone did not lead to changes in M170 amplitude, it was found to significantly interact with Prime/Target Match (β = 8.67, *t* = 2.60, *p* = 0.011). In matched prime-target trials (e.g., face → face), part-primes yielded significantly stronger amplitude than whole-primes (e.g., part-face → Mooney face > whole-face → Mooney face; *p* = 0.021, *d* = 0.61). This effect was not found for mismatched trials (*p* = 0.877; Figure 5 and Figure S5). Fifteen out of 20 participants showed the effect in the group direction (Figure S3). Although the regression analysis demonstrated that there was a moderate effect size for the difference between whole and part primes in matched trials (Cohen, 1988), it is not clear why the mean difference was not significant in the bootstrap analysis (Figure 6A). It is possible that we did not have enough power to achieve significance in that analysis.

Alternatively, amplitude from whole-prime trials was significantly stronger when the prime and target mismatched (e.g., whole-face prime → Mooney house > whole-face prime → Mooney face; *p* = 0.003, *d* = 0.42; Figure 5). This pairwise difference was also significant based on the bootstrap analysis (Figures 6B–D).

## Discussion

### The M100 discriminates faces from houses

A number of MEG and EEG studies have reported enhanced M100 or P1 amplitude for faces presented as photographs (e.g., Halgren et al., 2000; Liu et al., 2002; Itier and Taylor, 2004; Van Den Boomen et al., 2015) but not for faces presented as Mooneys (e.g., George et al., 2005; Latinus and Taylor, 2005, 2006). To our knowledge, our study is the first of its kind to demonstrate enhanced M100 amplitude for Mooney faces relative to Mooney houses. This effect was found for both tasks, although note that while the effect was not visible in the grand averaged, non-normalized data (in Figure S1), it was very clear and statistically robust in the normalized data (Figure 2).

Itier and Taylor (2004), who found a face-inversion effect at P1 with natural stimuli, proposed that P1 may index “global” sensitivity to face configuration that enables rapid distinction of faces from other objects. Mooney faces, which must be recognized holistically, could quite then plausibly elicit a face-house distinction at the M100. Although interpretation of the face-house difference in the passive data is fairly straightforward, the effect in the priming data requires some unpacking. *Post-hoc* tests determined that the effect was isolated to whole-prime trials. More specifically, amplitude was enhanced for Mooney houses only when preceded by a whole-face or a whole-house prime. This effect was only associated with the Mooney houses, as M100 amplitude was similar in response to Mooney faces preceded by a part prime or a whole prime. In line with interpretations from Itier and Taylor (2004), this observation, coupled with the lack of priming effects from prime-target compatibility (e.g., whether the target was preceded by a matching prime), supports conjecture that the M100 reflects a crude/preliminary stage of face perception based on detection of holistic information (e.g., Halgren et al., 2000).

### The M170 reflects holistic processing

The results of the M170 analyses support and supplement findings from previous MEG and EEG studies of face recognition. First, although visually underspecified, the M170 response to Mooney images was stronger for faces in the priming and passive tasks, and delayed for faces in the priming task. This result mirrors previous findings of differential M/N170 activation for faces (e.g., Halgren et al., 2000; Liu et al., 2000; Sagiv and Bentin, 2001; Latinus and Taylor, 2005).

Second, we found that compatibility (match) between the prime and target was associated with changes in M170 amplitude;, however, it was found to interact significantly with wholeness of the prime Trials with compatible (whole) primes were associated with significantly reduced M170 amplitude, consistent with expectancy-driven or prediction-based proposals. Specifically, M170 amplitude was reduced for Mooney faces preceded by whole face primes and for Mooney houses preceded by whole house primes.

Third, we found a distinction between holistic vs. component priming effects dependent on prime-target compatibility. Relative to component primes, holistic primes were associated with reduced M170 amplitude for compatible targets. Based on previous findings from repetition suppression (e.g., Harris and Nakayama, 2007, 2008), we interpret the whole-prime effect as facilitatory. We thus found a distinction between holistic vs. component priming for compatible vs. incompatible Mooney targets. The facilitatory effect of holistic faces and houses for Mooney face and houses, respectively, suggests that both Mooney face and house recognition is based on holistic information. There remain a number of issues regarding the interpretation of our results, which we discuss next.

The N170 component is implicated in processing top-down information given that it has been shown to be enhanced for famous but not non-famous faces (Jemel et al., 2003) as well as for primes from a different domain, such as a corresponding famous name (Jemel et al., 2005). It has been suggested that our finding of reduced M170 amplitude for matching whole primes could reflect top-down processing rather than holistic processing. This does not necessarily negate the importance of holistic information in face recognition. The holistic primes seem to offer an advantage over component primes in activating prior knowledge of the respective categories, suggesting holistic rather than component based processing. Our holistic priming effect adds to previous findings supporting the proposed link between face recognition and holistic processes (e.g., Richler et al., 2011a,b).

Our finding of facilitation from holistic primes on Mooney face recognition contradicts the view that holistic information does not seem to play a particularly important role in face recognition (e.g., Sekuler et al., 2004; Konar et al., 2010; Gold et al., 2012). As mentioned in the introduction, previous work has accounted for the face-inversion effect in quantitative rather than holistic vs. featural terms (Sekuler et al., 2004), finding that holistic processing is not correlated with recognition accuracy (Konar et al., 2010), and that whole faces offer no benefit over face components (Gold et al., 2012). Our results are difficult to compare with such findings because (a) our experiment was not designed to contrast inversion differences, (b) our experiment was not designed to measure recognition accuracy, and (c) we do not distinguish between the relative contribution of each of the different parts (e.g., mouth vs. eyes), so we cannot estimate whether the response to the face is predicted by the summed response to the wholes. These factors should be considered when designing future experiments. It would be interesting to test whether the advantage for whole primes would also be observed for Mooney targets to rule out quantitative explanations.

Also important to address are various issues concerning the degree of perceptual overlap between the part-primes and targets (amount of visual field with stimulus). It is not surprising that whole primes facilitated Mooney recognition. However, we found holistic priming of the M170 response *only* when the prime and target matched, suggesting that the effect is not explained in a simple manner by low-level visual information. Nevertheless, our study is limited because we do not know what the response profile of the M100 and M170 responses in our experiment would have been in *lieu* of holistic and component primes. As such, it is difficult to conclude unambiguously that reduced amplitude for targets preceded by holistic primes truly reflects facilitation. Additionally, it is very likely that the Mooney targets were more spatially matched to the components of the holistic primes than to the component primes, which always appeared in the center. It is also likely that the Mooney targets had greater similarity in power spectra to the holistic primes as compared to the component primes. It is possible then, that holistic priming effects were in part driven not just by the match between prime and target (face or house) but by the degree of match between the locations of the components with those of the target or by the greater correspondence in the power spectra. It is unlikely that the effects in this study were driven by spatial overlap, given that our experiment employed abstract Mooney images, which by definition do not have isolatable components. Nevertheless, future experiments should consider varying the degree of spatial overlap between primes and targets and equating the power spectra across categories of images (Torralba and Oliva, 2003; Crouzet and Thorpe, 2011). Of additional importance in future study is the contribution of individual features relative to the whole. This has been explored in previous studies involving faces presented as photographs (e.g., Sekuler et al., 2004; Konar et al., 2010; Gold et al., 2012) but not as Mooneys. Because our experiment did not distinguish between individual components (e.g., nose vs. eyes. vs. mouth) it was not possible to estimate whether facilitation from holistic primes was merely commensurate with the combined contribution of the individual components.

In sum, we found evidence of holistic processing during recognition of Mooney faces and houses. By exploiting the apparent cognitive penetrability of the M170 and employing Mooney images to maximize priming effects, we found that categorically compatible *holistic* information benefits recognition of ambiguous faces and houses. Moreover, this benefit is lost when the holistic information is incompatible with the ambiguous image. Together, these findings support the view of face recognition as involving holistic rather than featural processing at this processing stage.

## Author contributions

All authors listed, have made substantial, direct and intellectual contribution to the work, and approved it for publication.

### Conflict of interest statement

The authors declare that the research was conducted in the absence of any commercial or financial relationships that could be construed as a potential conflict of interest

## Footnotes

^1^The delay between presentation software timing and stimulus presentation (as measured with a photo-diode) was measured and informed time-stamping of data epochs.

## Supplementary material

Table S1 **Output from full model regressions**.

Figure S1 **(A)** Grand-average contour maps of neuromagnetic response distributions of M100 and M170 components in passive- (left) and priming-tasks (right). **(B)** Grand-average root-mean-squares (RMS) of normalized MEG activation (106 sensors) and standard error in passive and priming tasks.

Figure S2 **Pairwise difference comparison of the normalized M100 amplitude for Prime Wholeness and Target Interaction (see Figure 3 for data)**. Each bar represents the number of participants with a positive or negative difference in normalized M100 amplitude (amplitude for part prime trials minus amplitude for whole prime trials) for Mooney face targets and Mooney house targets. The normalized M100 amplitude for Mooney houses was found to be significantly greater in part prime trials than in whole prime trials (*p* = 0.005). This figure demonstrates that 15 out of 20 participants showed the effect in the group direction.

Figure S3 **Pairwise difference comparison of the normalized M170 amplitude for Prime Wholeness and Prime/Target Match (see Figure 4 for data)**. Each bar represents the number of participants with a positive or negative difference in normalized M170 amplitude (amplitude for part prime trials minus amplitude for whole prime trials) for Matched Prime/Target and Mismatched Prime/Target trials. The normalized M170 amplitude for matched prime-target trials was found to be significantly greater in part prime trials than in whole prime trials (*p* = 0.021). This figure demonstrates that 15 out of 20 participants showed the effect in the group direction.

Figure S4 **Plots for M100 Amplitude interaction between Prime-Wholeness and Target (^*^*p* < 0.05)**.

Figure S5 **Plots for M170 Amplitude interaction between Prime-Wholeness and Prime-Target-Match (^*^*p* < 0.05)**.

Figure S6 **The grand average of the eight experimental conditions in the priming task**. Each plot shows the grand mean of the normalized RMS data across subjects and the shaded SEM.

Figure S7 **Comparisons of the major contrasts of the normalized RMS MEG data**. In each plot, the shaded area represents the bootstrapped standard error. The thick black line corresponds to the first condition in the title and the thin black line to the second condition. The standard error was computed with a percentile bootstrap (1000 samples) with replacement at *p* < 0.05.

Figure S8 **Comparisons of the major contrasts of the normalized RMS MEG data**. In each plot, the shaded area represents the confidence interval. The thick black line corresponds to the first condition in the title and the thin black line to the second condition. The confidence intervals were computed with a percentile bootstrap (1000 samples) with replacement at *p* < 0.05. The red line indicates that the confidence interval did not include zero.
