# Supplementary material for: Effects of Part- and Whole-Object Primes on Early MEG Responses to Mooney Faces and Houses
Source: Front Psychol. 2016 Feb 16;7:147. doi: 10.3389/fpsyg.2016.00147 (PMC4754396; doi:10.3389/fpsyg.2016.00147)
Supplement: Table S1 — Output from full model regressions. [file Table1.docx]

Table 1: Full model output from final regression models (Steinberg Lowe, Lewis & Poeppel)
