# Supplementary figures and images for: Effects of Part- and Whole-Object Primes on Early MEG Responses to Mooney Faces and Houses
Source: Front Psychol. 2016 Feb 16;7:147. doi: 10.3389/fpsyg.2016.00147 (PMC4754396; doi:10.3389/fpsyg.2016.00147)

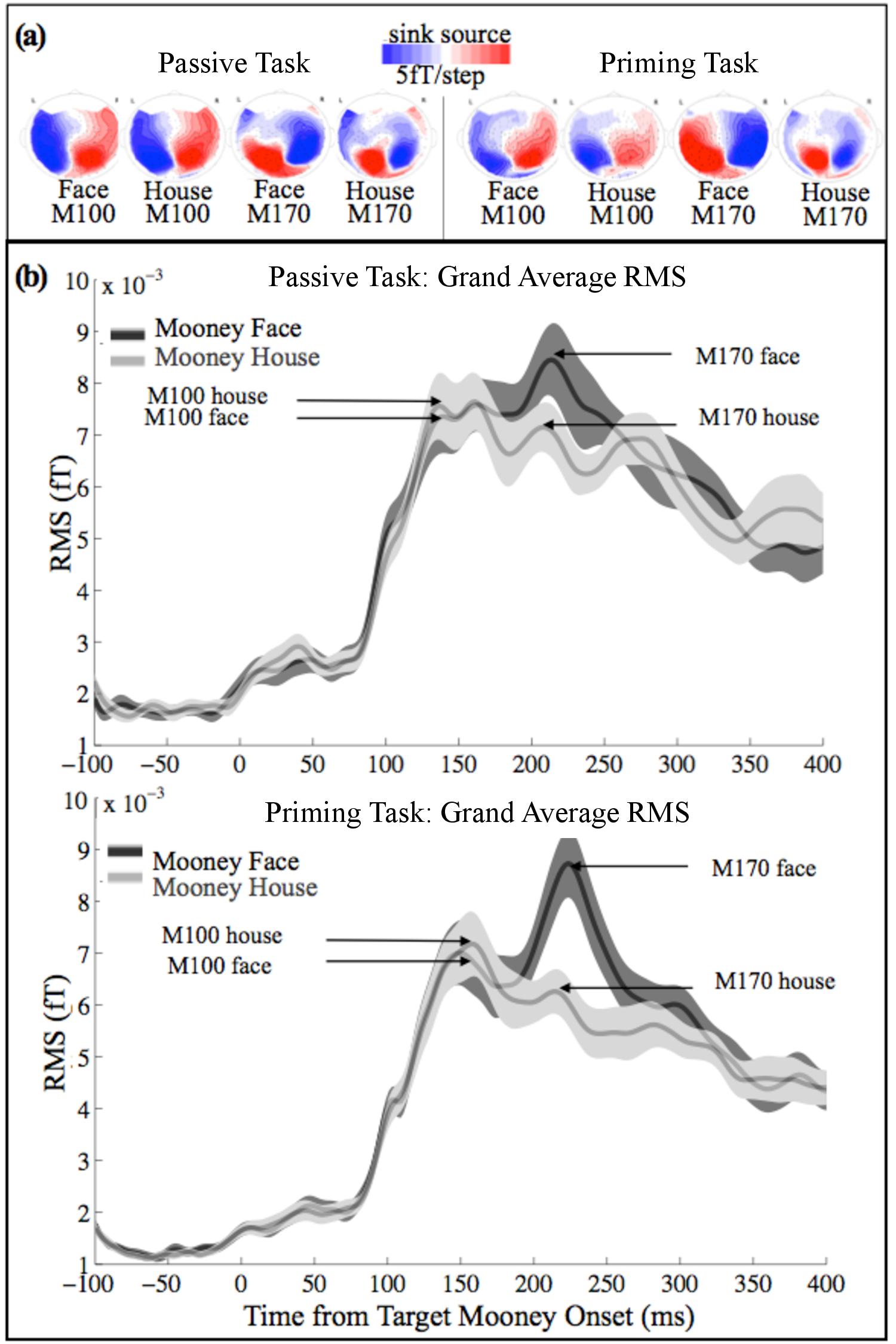

Supplement: Figure S1 — (A) Grand-average contour maps of neuromagnetic response distributions of M100 and M170 components in passive- (left) and priming-tasks (right). (B) Grand-average root-mean-squares (RMS) of normalized MEG activation (106 sensors) and standard error in passive and priming tasks. [file Image1.JPEG]

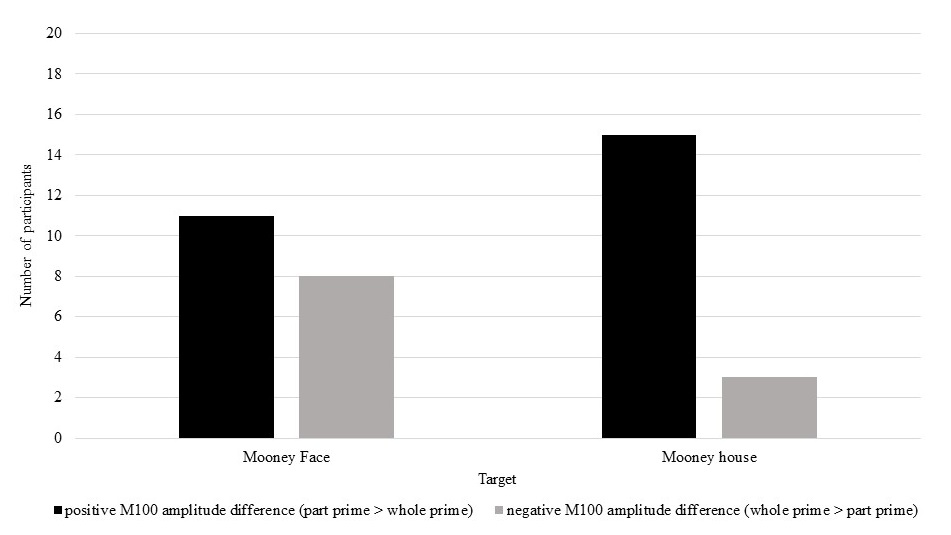

Supplement: Figure S2 — Pairwise difference comparison of the normalized M100 amplitude for Prime Wholeness and Target Interaction (see Figure 3 for data). Each bar represents the number of participants with a positive or negative difference in normalized M100 amplitude (amplitude for part prime trials minus amplitude for whole prime trials) for Mooney face targets and Mooney house targets. The normalized M100 amplitude for Mooney houses was found to be significantly greater in part prime trials than in whole prime trials (p = 0.005). This figure demonstrates that 15 out of 20 participants showed the effect in the group direction. [file Image2.JPEG]

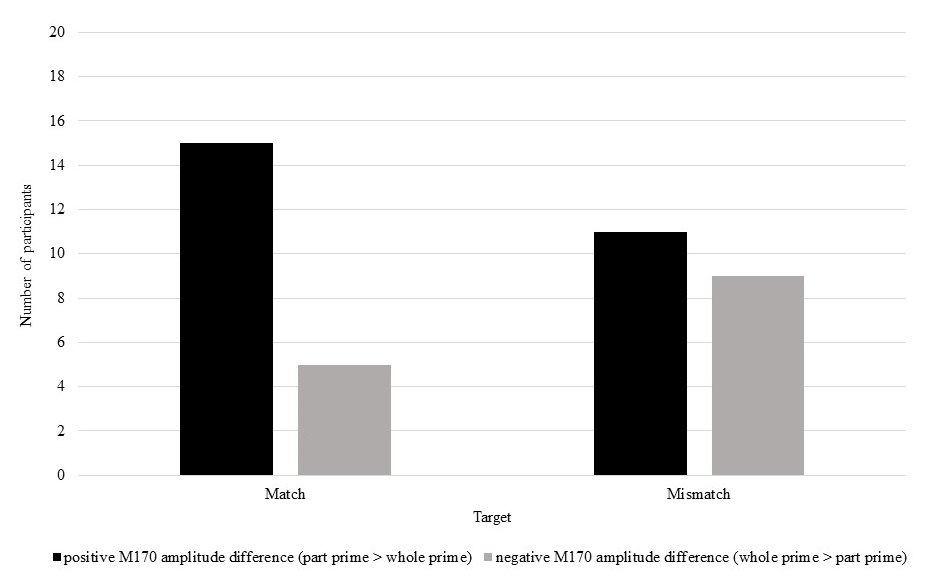

Supplement: Figure S3 — Pairwise difference comparison of the normalized M170 amplitude for Prime Wholeness and Prime/Target Match (see Figure 4 for data). Each bar represents the number of participants with a positive or negative difference in normalized M170 amplitude (amplitude for part prime trials minus amplitude for whole prime trials) for Matched Prime/Target and Mismatched Prime/Target trials. The normalized M170 amplitude for matched prime-target trials was found to be significantly greater in part prime trials than in whole prime trials (p = 0.021). This figure demonstrates that 15 out of 20 participants showed the effect in the group direction. [file Image3.JPEG]

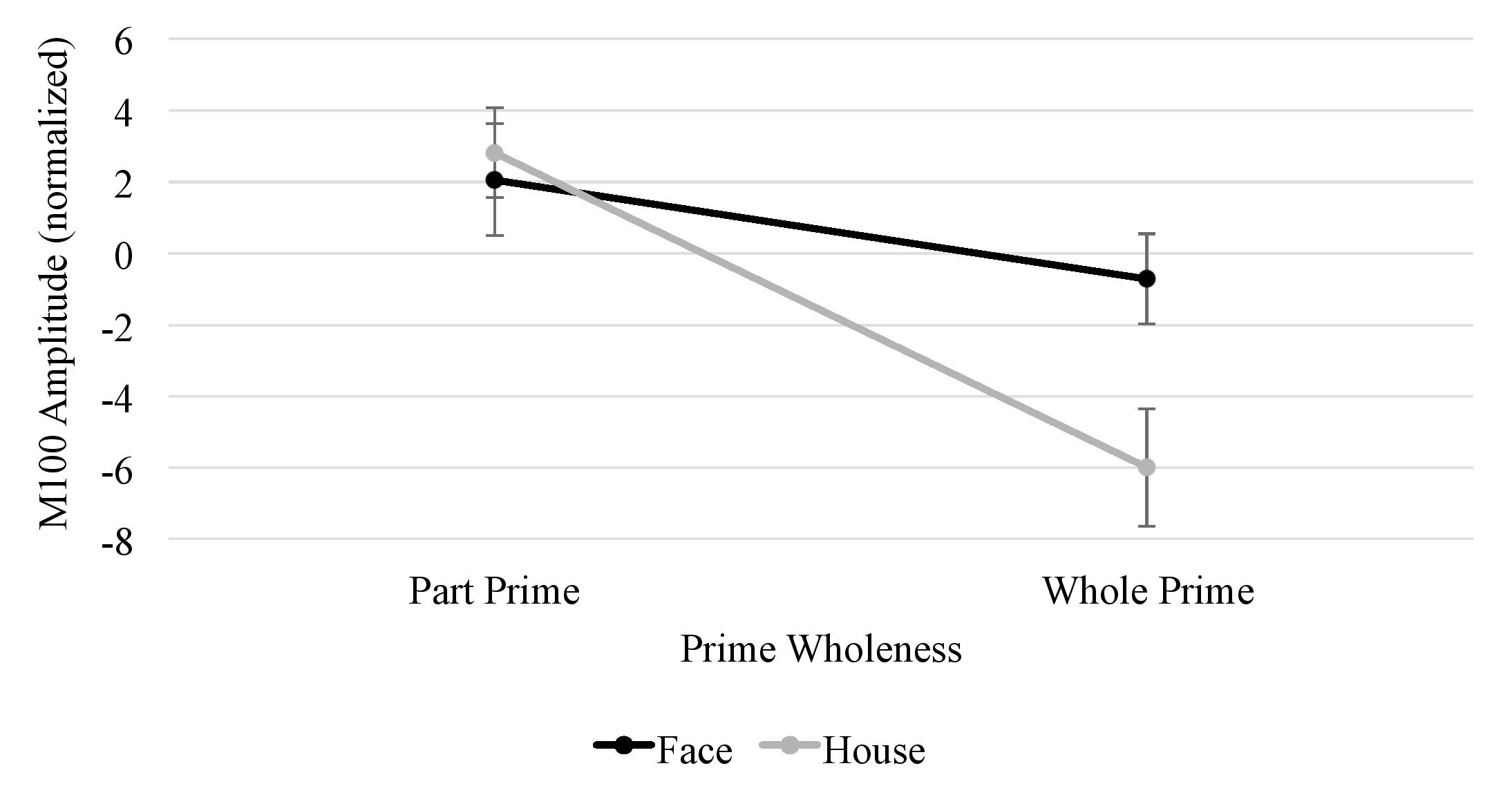

Supplement: Figure S4 — Plots for M100 Amplitude interaction between Prime-Wholeness and Target (*p < 0.05). [file Image4.JPEG]

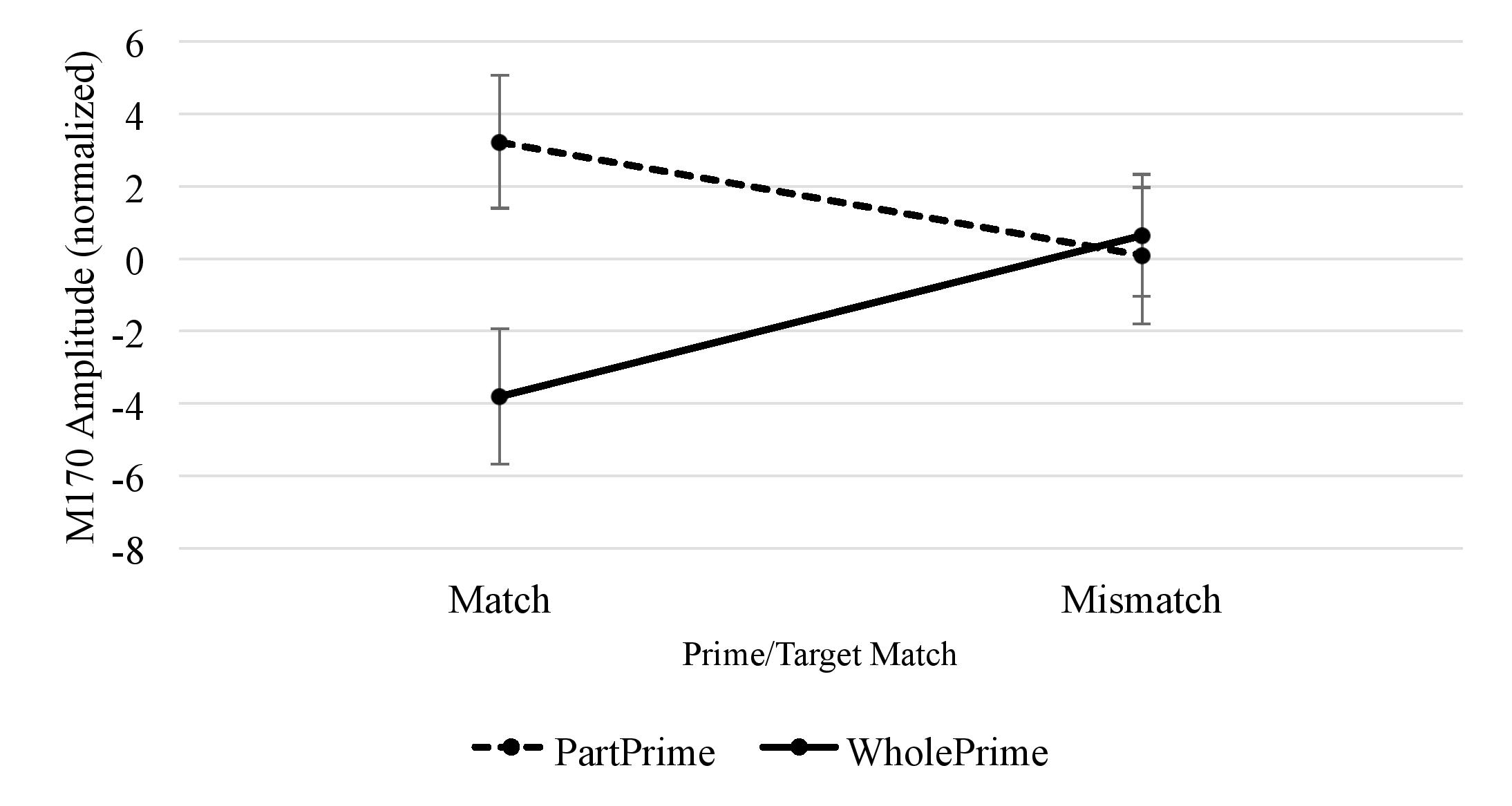

Supplement: Figure S5 — Plots for M170 Amplitude interaction between Prime-Wholeness and Prime-Target-Match (*p < 0.05). [file Image5.JPEG]

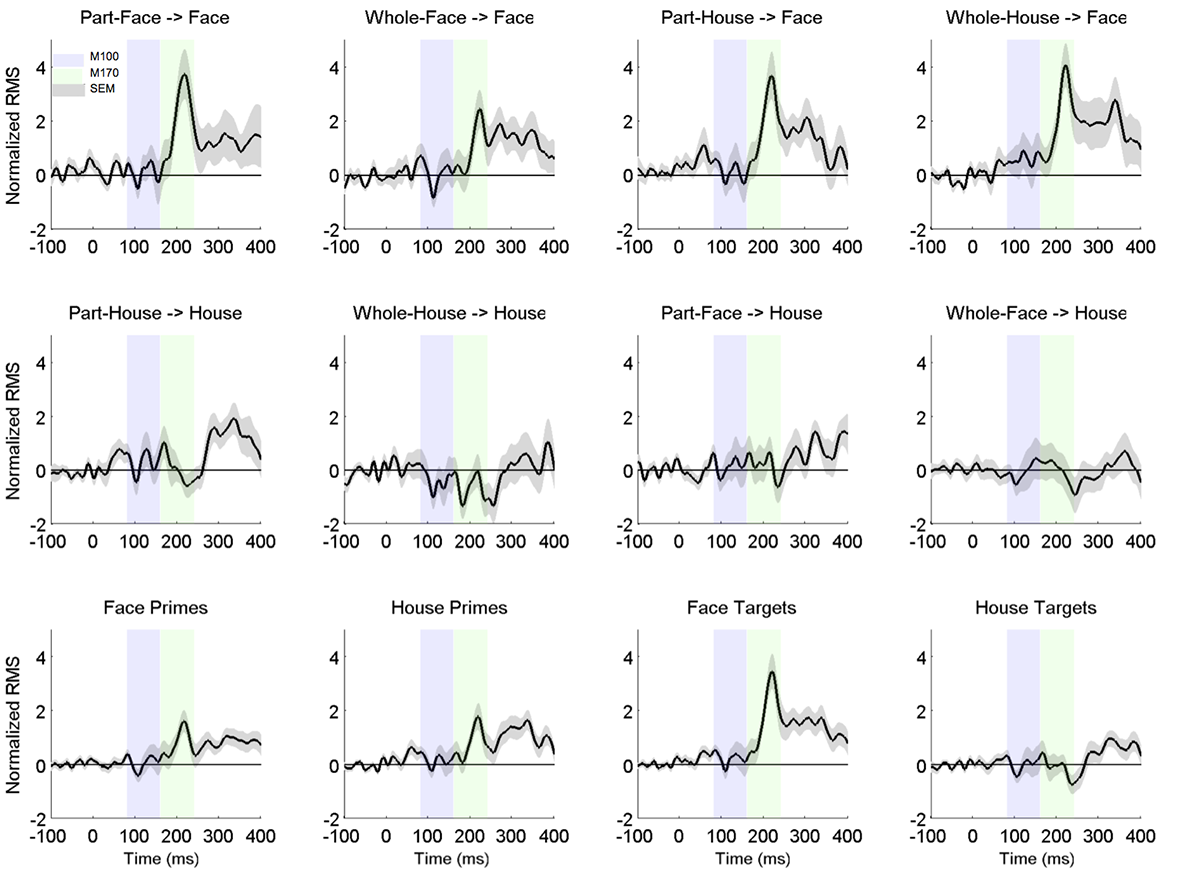

Supplement: Figure S6 — The grand average of the eight experimental conditions in the priming task. Each plot shows the grand mean of the normalized RMS data across subjects and the shaded SEM. [file Image6.TIF]

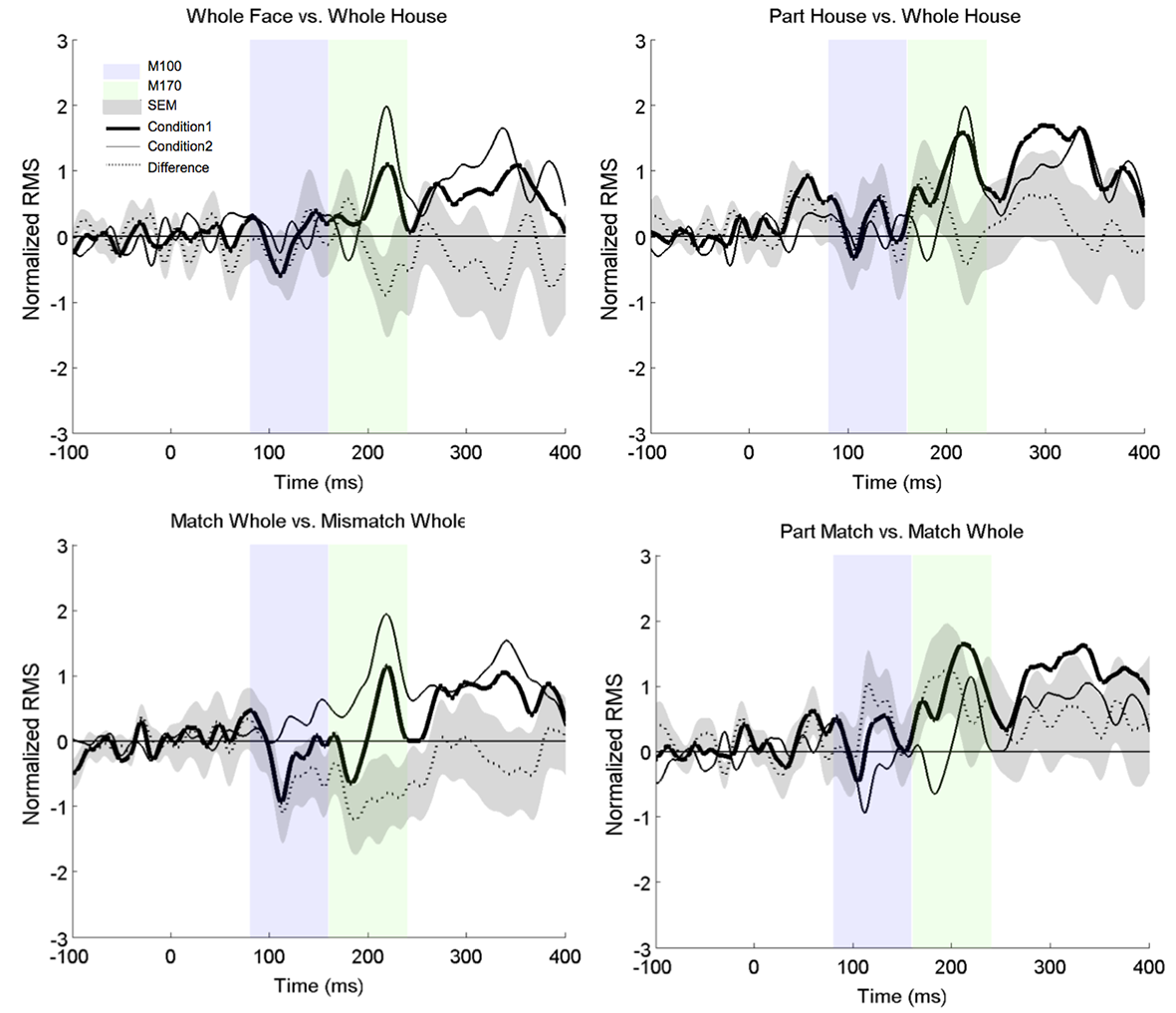

Supplement: Figure S7 — Comparisons of the major contrasts of the normalized RMS MEG data. In each plot, the shaded area represents the bootstrapped standard error. The thick black line corresponds to the first condition in the title and the thin black line to the second condition. The standard error was computed with a percentile bootstrap (1000 samples) with replacement at p < 0.05. [file Image7.TIF]

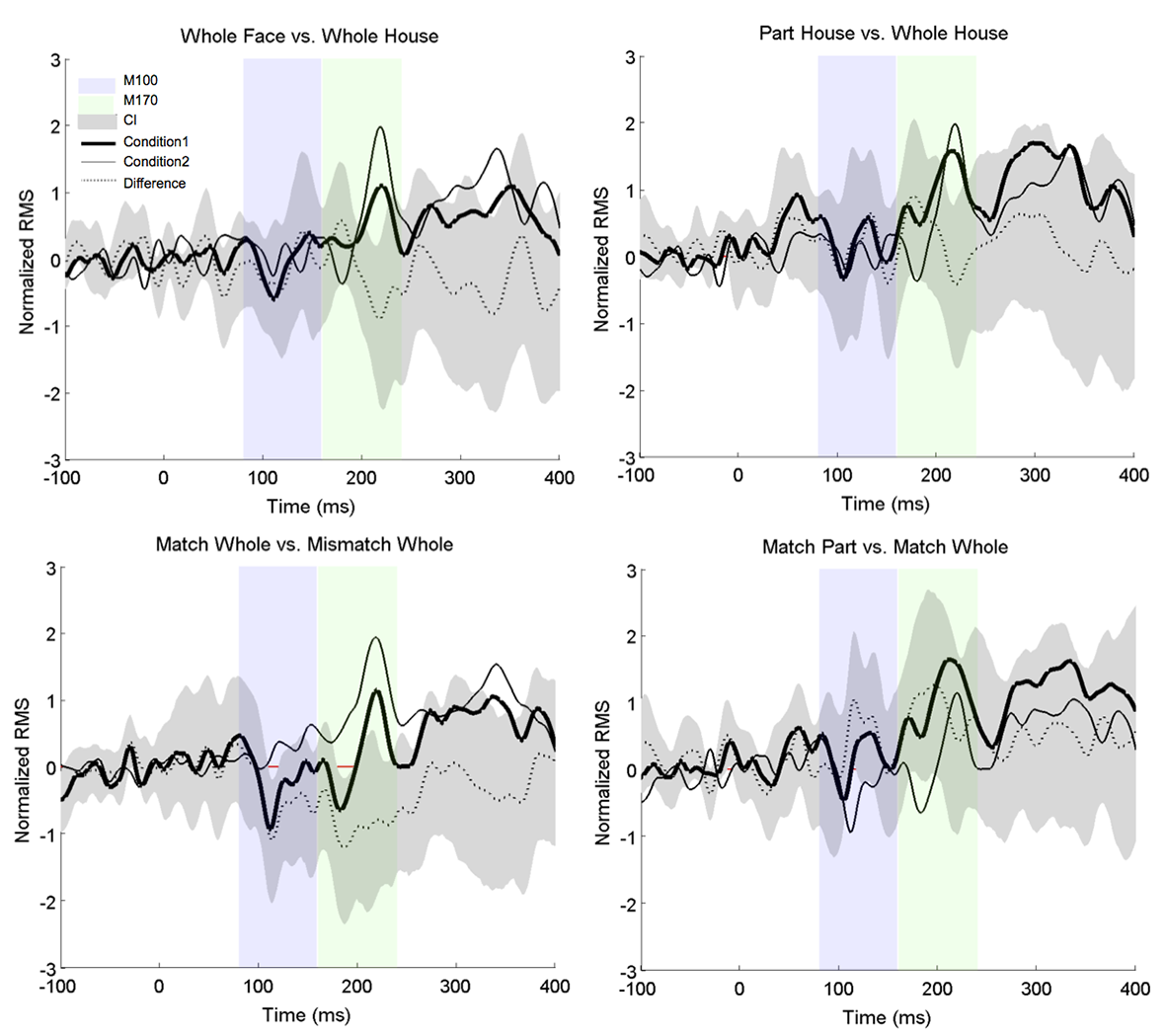

Supplement: Figure S8 — Comparisons of the major contrasts of the normalized RMS MEG data. In each plot, the shaded area represents the confidence interval. The thick black line corresponds to the first condition in the title and the thin black line to the second condition. The confidence intervals were computed with a percentile bootstrap (1000 samples) with replacement at p < 0.05. The red line indicates that the confidence interval did not include zero. [file Image8.TIFF]
